# Formyl-Peptide Receptor 2 Signaling Redirects Glucose and Glutamine into Anabolic Pathways in Metabolic Reprogramming of Lung Cancer Cells

**DOI:** 10.3390/antiox11091692

**Published:** 2022-08-29

**Authors:** Tiziana Pecchillo Cimmino, Ester Pagano, Mariano Stornaiuolo, Gabriella Esposito, Rosario Ammendola, Fabio Cattaneo

**Affiliations:** 1 Department of Molecular Medicine and Medical Biotechnology, School of Medicine, University of Naples Federico II, 80131 Naples, Italy; 2 Department of Pharmacy, School of Medicine, University of Naples Federico II, 80131 Naples, Italy

**Keywords:** formyl-peptide receptors, NADPH oxidase, metabolic reprogramming, glucose metabolism, glutamine transporter, synthesis of pyrimidine nucleotides

## Abstract

Glucose and glutamine play a crucial role in the metabolic reprogramming of cancer cells. Proliferating cells metabolize glucose in the aerobic glycolysis for energy supply, and glucose and glutamine represent the primary sources of carbon atoms for the biosynthesis of nucleotides, amino acids, and lipids. Glutamine is also an important nitrogen donor for the production of nucleotides, amino acids, and nicotinamide. Several membrane receptors strictly control metabolic reprogramming in cancer cells and are considered new potential therapeutic targets. Formyl-peptide receptor 2 (FPR2) belongs to a small family of GPCRs and is implicated in many physiopathological processes. Its stimulation induces, among other things, NADPH oxidase-dependent ROS generation that, in turn, contributes to intracellular signaling. Previously, by phosphoproteomic analysis, we observed that numerous proteins involved in energetic metabolism are uniquely phosphorylated upon FPR2 stimulation. Herein, we investigated the role of FPR2 in cell metabolism, and we observed that the concentrations of several metabolites associated with the pentose phosphate pathway (PPP), tricarboxylic acid cycle, nucleotide synthesis, and glutamine metabolism, were significantly enhanced in FPR2-stimulated cells. In particular, we found that the binding of specific FPR2 agonists: (i) promotes NADPH production; (ii) activates the non-oxidative phase of PPP; (iii) induces the expression of the ASCT2 glutamine transporter; (iv) regulates oxidative phosphorylation; and (v) induces the de novo synthesis of pyrimidine nucleotides, which requires FPR2-dependent ROS generation.

## 1. Introduction

Metabolic reprogramming sustains the cell growth and proliferation of cancer cells through the regulation of energy metabolism [1]. Normal cells obtain energy via cytosolic glycolysis followed by mitochondrial oxidative phosphorylation (OXPHOS) under aerobic conditions or mainly via glycolysis under anaerobic conditions. Cancer cells prefer to perform glycolysis, even in normoxic conditions, a phenomenon known as the “Warburg effect” or “aerobic glycolysis” [2,3]. In this process, the efficiency of ATP production per molecule of glucose is much lower, but the yield rate is much faster than that in OXPHOS [4]. Therefore, a high rate of glucose metabolism is required to meet the increased energy demand that supports the fast growth and proliferation of cancer cells. Furthermore, the enhancement of aerobic glycolysis in tumors provides more carbon intermediates for the biosynthesis of nucleotides, amino acids, and lipids; mitochondrial biogenesis; and other bioenergetic metabolic pathways [5,6,7]. Glycolysis, the central pathway of glucose metabolism, can be branched to the pentose phosphate pathway (PPP) via glucose-6P, to the serine synthesis pathway via 3-phosphoglicerate, and to the hexosamine biosynthetic pathway via fructose-6-phosphate, which provides UDP-N-acetylglucosamine. This metabolite is an activated monosaccharide that is used as a substrate by glycosyltrasferases in glycosylation reactions of proteins and lipids but also coordinates growth-factor-induced glucose and glutamine metabolism [8]. In cancer cells, glucose and glutamine represent the primary sources of carbon atoms for biosynthesis as well as for cancer cell survival and proliferation because they feed glycolysis and the tricarboxylic acid cycle (TCA). Glutamine is also an important nitrogen donor for the production of nucleotides, amino acids, and nicotinamide.

Several membrane receptors strictly control metabolic reprogramming in cancer cells. Some tyrosine kinase receptors (TKRs) act by suppressing pyruvate dehydrogenase complex (PDC) activity via the direct phosphorylation of tyrosine residues [9]. In cancers, the upregulation of HER4, a member of the epidermal growth factor receptor (EGFR) family, increases metabolic processes associated with tumor promotion, including glycolysis, OXPHOS, and glutaminolysis [10]. Other examples of receptors involved in regulating metabolic reprogramming include Toll-like receptor 4 [11], C-C chemokine receptor type 7 [12], and the nuclear receptor (NR) superfamily of transcription factors [13].

Formyl peptide receptors (FPRs) belong to a small family of G-protein-coupled receptors (GPCRs), and three members of this family, FPR1, FPR2, and FPR3, have been identified in humans. They have been implicated in many physiopathological processes, such as neurodegeneration [14,15] and cancer [16,17,18]. FPRs are primarily expressed in the innate and adaptive immune systems, where they contribute to the detection and elimination of bacterial pathogens through the recognition of pathogen-associated molecular patterns and damage-associated molecular patterns [19]. FPR2 is expressed onto the plasma membranes of various other cell types [20,21,22] and onto the nuclear membranes of some eukaryotic cells [23]. It is the most promiscuous member of the FPR family because it is activated by a large number of ligands, including the synthetic peptide WKYMVm, annexin A1 (ANXA1), a phospholipid-binding protein widely expressed in many tissues, and lipoxin A4 (LXA4), an endogenous lipoxygenase-derived eicosanoid mediator [24]. The binding of WKYMVm, ANXA1, and LXA4 to FPR2 triggers anti-inflammatory responses [25,26,27], whereas serum amyloid alpha (SAA) acts as a pro-inflammatory agonist on FPR2 [28]. The conformational changes of FPR2 upon ligand binding are responsible for the switch between the anti-inflammatory and pro-inflammatory responses [29].

Following FPR2 stimulation, several protein kinases are activated and a large number of signaling and non-signaling proteins are phosphorylated, [20,21,30], including the cytosolic subunits p47^phox^ and p67^phox^ of NADPH oxidase, whose phosphorylation is required for NADPH oxidase-dependent reactive oxygen species (ROS) generation [31,32]. The responses to stimuli of many receptors include integrated networks of complex signals that operate coordinately. Their control is exerted through the “phosphorylation/dephosphorylation” switch catalyzed by protein kinases and phosphatases (PTPases) [33,34]. A dysfunction of the synergistic action between protein kinases and PTPases is responsible for several human diseases. In FPR2-stimulated cells, several phosphosites, identified in both protein kinases and PTPases, play critical functions in the molecular mechanisms of regulation or in the fine-tuning of switch properties [35].

TKR transactivation, mediated by some members of the GPCR family, constitutes a molecular mechanism used by many cell types to expand the amount and spectrum of signaling networks [36]. We previously demonstrated that FPR1 and FPR2 stimulation induced ROS-dependent TKR transactivation and, in turn, the activation of peculiar intracellular signaling pathways triggered by TKRs [32,37,38,39,40]. In addition, we carried out a phosphoproteomic analysis demonstrating that, in the human CaLu-6 epithelial carcinoma cell line, FPR2 stimulation with WKYMVm or ANXA1 induced the redox-regulated phosphorylation of several proteins, most of which participated in different aspects of cellular metabolic processes, including primary metabolism [30,35]. Therefore, we herein utilize a metabolomic approach to identify the metabolic pathways activated in WKYMVm- and ANXA1-stimulated CaLu-6 cells. 

## 2. Materials and Methods

### 2.1. Cell Culture and Reagents

Human lung cancer CaLu-6 cells (ATTC, Manassas, VA, USA) and p22phoxCrispr/Cas9 CaLu-6 cells were grown in Dulbecco’s modified Eagle’s medium (DMEM), supplied with 10% fetal bovine serum (FBS) (Invitrogen Corp., Carlsbad, CA, USA), at 37 °C and 5% CO_2_ until 70% confluency and serum-starved for 24 h. Growth-arrested CaLu-6 cells were stimulated or not with 10 μM WKYMVm (Primm, Milan, Italy) or 10 nM annexin A1 (ANXA1) (Bio-Techne, Minneapolis, MN, USA) for various times. In other experiments, cells were preincubated with WRWWWW (WRW4) (Primm, Milan, Italy) for 15 min at a final concentration of 10 μM or with apocynin (Sigma Chemical, St. Louis, MO, USA) for 2 h at a final concentration of 5 mM and stimulated or not with WKYMVm or ANXA1.

### 2.2. Metabolomic Analysis by LC-MS

A metabolomic analysis by LC-MS was performed in CaLu-6 cells stimulated or not with WKYMVm in the presence or absence of WRW4. Briefly, 2 × 10^4^ cells were plated in a 48-multiwell plate and the day after were serum-starved for 24 h before the treatments. Cell monolayers were rinsed in cold water to then be lysed in 400 μL of a 1:1 prechilled MetOH/H_2_O solution. The samples were vortex-mixed, kept on ice for 20 min, and centrifuged again at 10,000× *g* at 4 °C for 10 min. The collected supernatant was dried in a SpeedVac concentrator system (Thermo Scientific, Waltham, MA, USA) operated at room temperature. Dried supernatants were reconstituted with 125 μL of methanol/acetonitrile/water (50:25:25). The extracted metabolites were analyzed using an ACQUITY UPLC system online-coupled to a Synapt G2-Si QTOF-MS (Waters Corporation, Milford, MA, USA) in positive and negative modes in the following settings: reverse-phase ACQUITY UPLC CSH C18 (1.7-μm, 100 × 2.1 mm^2^) column (Waters), 0.3 mL/min flow rate, mobile phases composed of acetonitrile/H20 (60:40) containing 0.1% formic acid and 10 mM ammonium formate (Phase A), and isopropanol/acetonitrile (90:10) containing 0.1% formic acid and 10 mM ammonium formate (Phase B). Peak detection, metabolite identification, and quantitation were performed as previously described [41], fitting experimental data with internal standard and calibration curves. Data analysis was performed and a heatmap was generated with the online software MetaboAnalyst (https://www.metaboanalyst.ca, accessed on 1 June 2021), as previously reported [42,43].

### 2.3. p22phoxCrispr/Cas9 Double-Nickase CaLu-6 Cells

p22phoxCrispr/Cas9 cells were generated by transfecting CaLu-6 cells with a Double Nickase Plasmid or with a control Double Nickase Plasmid (Santa Cruz Biotechnology, Irvine, CA, USA), as previously described [20]. Puromycin-positive selection was performed to isolate p22phoxCrispr/Cas9 CaLu-6 cells, and p22phox expression was tested by Western blotting. p22phox knockout clones were collected in order to obtain p22phoxCrispr/Cas9 CaLu-6 cells.

### 2.4. Protein Extraction and Western Blot

Whole protein lysates were purified from 24 h serum-starved CaLu-6 or p22phoxCrispr/Cas9 CaLu-6 cells stimulated or not with 10 μM WKYMVm or 10 nM ANXA1 in the presence or absence of the above-mentioned selective inhibitors. Whole lysates were obtained as previously described [44] by scraping cells with ice cold RIPA buffer containing: 50 mM Tris–HCl, pH 7.4, 150 mM NaCl, 1% NP-40, 1 mM EDTA, 0.25% sodium deoxycholate, 1 mM NaF, 10 μM Na_3_VO_4_, 1 mM phenylmethylsulfonylfluoride, 10 μg/mL aprotinin, 10 μg/mL pepstatin, and 10 μg/mL leupeptin. A Bio-Rad protein assay was used to determine protein concentrations (BioRAD, Hercules, CA, USA). A Western blot analysis was performed as previously described [45].

Anti-GAPDH (SC-47724), anti-tubulin (SC-53646), and anti-phospho-c-Myc (Ser62) (SC-8000-R) were purchased from Santa Cruz Biotechnology (Irvine, CA, USA). Anti-phospho-CAD (Ser 1859) and anti-ASCT2 were purchased from Cell Signalling Technology (Denvers, MA, USA).

Proteins were visualized by an enhanced chemiluminescence reagent (Amersham Biosciences, Little Chalfont, Buckinghamshire, UK) and were quantified using densitometry (Chemidoc, Bio-Rad). Each experiment and densitometric quantification were separately repeated at least three times.

### 2.5. Mitochondrial Membrane Potential Assay

The mitochondrial membrane potential (Δψm) was assessed in CaLu-6 cells with MitoTracker^®^ Red CMXRos (Thermo Fisher Scientific) dye according to the manufacturer’s instructions. Briefly, CaLu-6 cells were rinsed twice in PBS before adding the dye. Cells were incubated in the presence of the probe for 45 min at 37 °C and 5% CO_2_. Thereafter, cells were rinsed three times in DMEM and once in PBS, fixed in 3.7% formaldehyde for 30 min, permeabilized in 0.1% Triton X-100 in PBS, and stained with the nuclear dye DAPI. Mitochondrial fluorescence was measured in a Perkin Elmer Envision 2105 Multiplate reader (Perkin Elmer) using the built-in monochromator and the following parameters: λ excitation 579 nm, λ emission 599 nm for MitoTracker, λ excitation 351 nm, and λ emission 450 nm for DAPI. The total number of cells in each well was used for normalization. The results are the means of three independent experiments, and in each separate experiment, every experimental point was analyzed in triplicate.

### 2.6. NADP^+^/NADPH Assay

An NADP^+^/NADPH assay was performed according to manufacturer’s instruction (Elabscience, Houston, TX, USA) to calculate NADP+, NADPH, and their ratio. Briefly, CaLu-6 cells were serum-starved for 24 h and then stimulated or not with 10 μM WKYMVm or 10 nM ANXA1 for different times. In other experiments, serum-starved cells were pretreated with WRW4 and stimulated or not with WKYMVm or ANXA1. NADP^+^ and NADPH were quantified in a colorimetric assay by measuring the OD value at 450 nm. The results are the means of three independent experiments, and in each separate experiment, every experimental point was analyzed in triplicate.

### 2.7. Transketolase Activity Assay

Transketolase (TKT) enzymatic activity was measured in CaLu-6 cells by a Transketolase activity assay kit (Sigma-Aldrich, Saint Louis, MO, USA), following the manufacturer’s instructions. Briefly, 4 × 10^5^ CaLu-6 cells were serum-starved for 24 h and stimulated with 10 μM WKYMVm or 10 nM ANXA1 for different times. In other experiments, serum-starved CaLu-6 cells were pretreated with WRW4 and then stimulated with WKYMVm or ANXA1. For each sample, 8 μg of cell lysates were added into each well of a 96-well plate, and TKT activity was determined by recording the fluorescence (RFU) released from the conversion of a non-fluorescent probe to a fluorescent probe (λEx = 535/λEm = 587). All samples and standards were run in duplicate. The results are the means of three independent experiments.

### 2.8. Statistical Analysis

An unpaired *t*-test was used to compare the means of two independent groups of experiments; a one-way analysis of variance (ANOVA) was used to compare more than two groups of experiments. GraphPad Prism 7 was used for statistical analysis (GraphPad Software Inc., San Diego, CA, USA). All reported data are representative of at least three independent experiments and are expressed as means ± the standard error of the mean (SEM). A *p* value of less than 0.05 was considered to be statistically significant.

## 3. Results and Discussion

### 3.1. FPR2 Stimulation Triggers PPP

**Figure 1:**
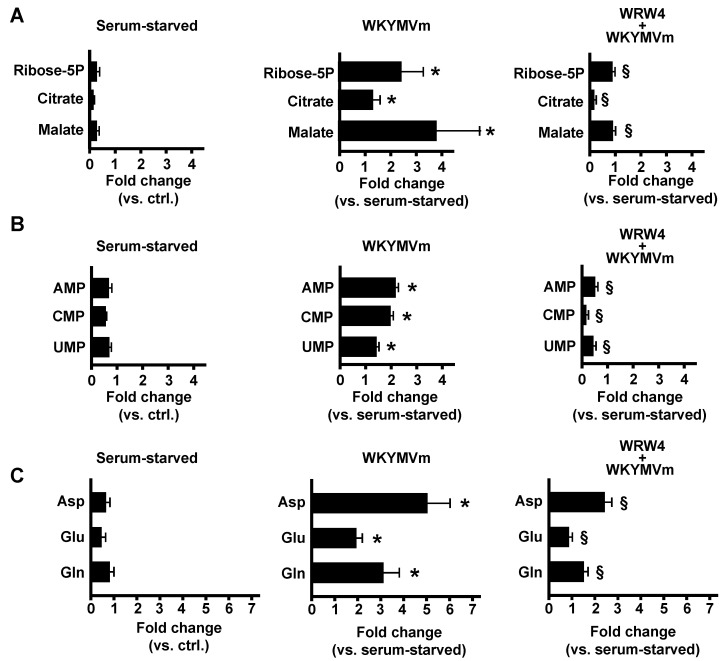
**FPR2 stimulation modulates lung cancer cell metabolism.** Growing cells (ctrl.) were serum-starved for 24 h and then stimulated or not with 10 μM WKYMVm for 1 h in the presence or absence of 10 μM WRWWWW (WRW4). The metabolomic analysis was performed as described in the Materials and Methods Section. The FPR2-dependent modulation of metabolites involved in glucose metabolism, nucleotide synthesis, and amino acid metabolism is reported in Panels (**A**,**B**,**C**), respectively. * *p* < 0.05 compared to unstimulated cells. § *p* < 0.05 compared to stimulated cells. Ribose-5P: ribose 5-phosphate; Asp: aspartate; Glu: glutamate; Gln: glutamine.

We analyzed the metabolic response of WKYMVm-stimulated CaLu-6 cells. In comparison to untreated cells, stimulated cells displayed increased concentrations of ribose 5-phosphate (Ribose-5P), citrate, and malate, which was consistent with the activation of glucose oxidation via PPP and TCA (Figure 1A). Preincubation with an FPR2 antagonist, the peptide WRW4, prevented this increase (Figure 1A).

PPP branches from glycolysis at the first committed step of glucose metabolism catalyzed by hexokinase and consumes glucose-6-phosphate (G6P) as a primary substrate. The PPP consists of both oxidative and non-oxidative phases. The oxidative phase generates NADPH and Ribose-5P in three irreversible reactions. In the first of these, G6P is dehydrogenated by G6P dehydrogenase (G6PDH) to yield NADPH and 6-phosphogluconlactone, which is subsequently hydrolyzed by phosphogluconolactonase into 6-phosphogluconate. The third irreversible reaction is the oxidative decarboxylation of 6-phosphogluconate to yield a second molecule of NADPH and ribulose-5-phosphate, which is then converted to Ribose-5P, a precursor of nucleotide biosynthesis. Therefore, PPP is critical for cancer cells because it generates pentose phosphates to supply their high rate of nucleic acid synthesis and NADPH, which is required for reductive biosynthesis and to maintain redox balance under stress situations when cells proliferate rapidly [46]. In agreement, in FPR2-stimulated CaLu-6 cells, our metabolomic analysis also revealed enhanced concentrations of AMP, CMP, and UMP, which were prevented by preincubation with WRW4 (Figure 1B). 

The pyrimidine rings of nucleotides are synthesized de novo as uracil from aspartate, CO_2_, and glutamine. Aspartate provides three of the four carbon atoms and one nitrogen atom. CO_2_ provides the fourth carbon atoms, and the second nitrogen atom is supplied by glutamine. For the synthesis of purine rings of nucleotides, five carbon atoms derive from CO_2_, glycine, and from one carbon unit of N_10_-formyl-TetraHydroFolate (THF), which is derived from the serine–glycine pathway via N_5_, N_10_-methylene-THF. Aspartate provides one nitrogen atom, while serine and glycine may be derived, via de novo synthesis, from 3-phosphoglicerate [47]. Notably, our metabolomic analysis revealed FPR2-dependent increases in the aspartate, glutamate, and glutamine concentrations in WKYMVm-stimulated CaLu-6 cells (Figure 1C).

### 3.2. NADPH Production and the Non-Oxidative Phase of PPP Are Regulated by FPR2

**Figure 2:**
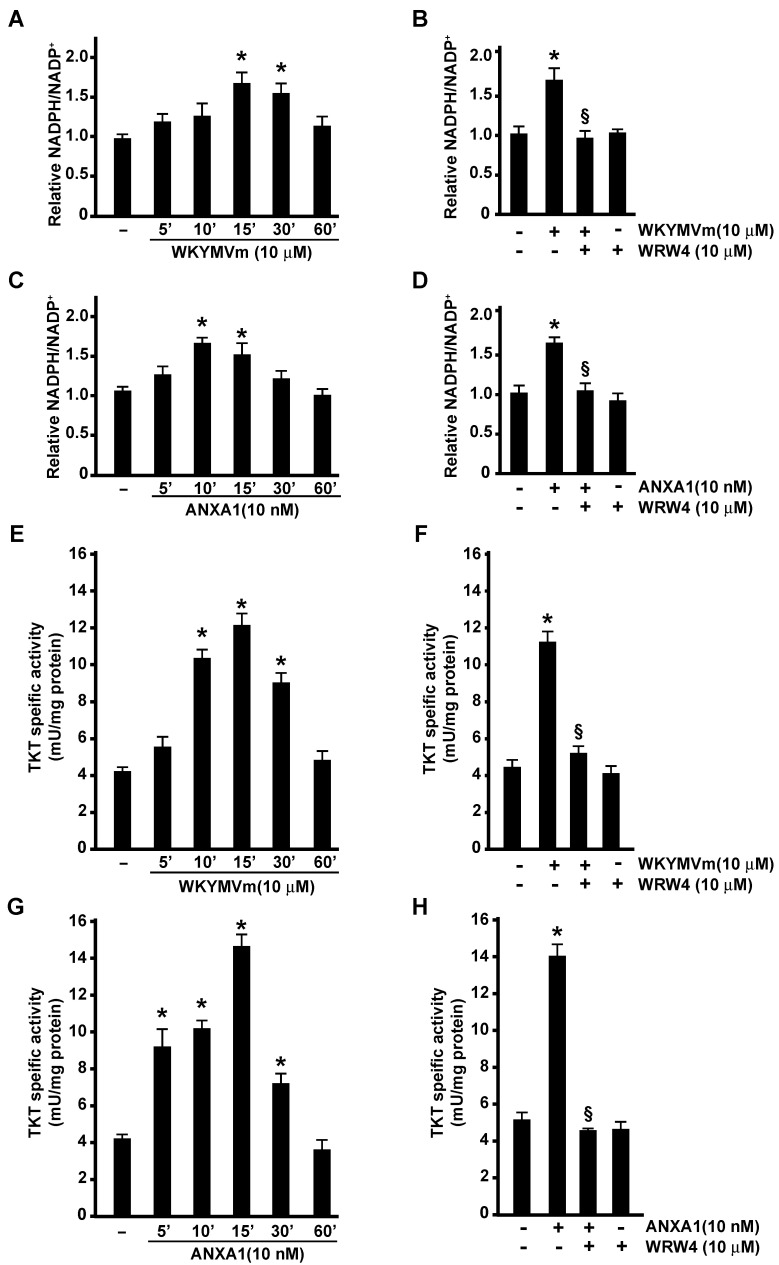
**FPR2 stimulation enhances NADPH production and regulates non-oxidative phase of pentose phosphate pathway.** CaLu-6 cells were serum-starved for 24 h and then stimulated with WKYMVm (Panels (**A**,**E**)) or ANXA1 (Panels (**C**,**G**)) for the indicated times. Cells were also preincubated with WRW4 before WKYMVm (Panels (**B**,**F**)) or ANXA1 (Panels (**D**,**H**)) stimulation. NADPH/NADP+ assay was performed according to manufacturer’s instruction (Panels (**A**–**D**)). Transketolase (TKT) enzymatic activity was determined by recording fluorescence (RFU) released from the conversion of non-fluorescent probe to a fluorescent probe (λEx = 535/λEm = 587). Data are reported in bar graphs (Panels (**E**–**H**)). Data are representative of three independent experiments. * *p* < 0.05 compared to unstimulated cells. § *p* < 0.05 compared to stimulated cells.

NADPH is one of the products generated in the PPP, and in proliferating cells, the largest contributor to NADPH is the oxidative PPP. Therefore, we measured NADPH production in FPR2-stimulated cells, and we found that either WKYMVm or ANXA1 induced a time-dependent increase in NADPH (Figure 2A,C) that was prevented by WRW4 (Figure 2B,D).

In neutrophils and in several cell types, both FPR2 agonists also trigger NADPH oxidase-dependent ROS production [20,32,48,49,50,51] that depends upon a constant source of intracellular NADPH. Indeed, during oxidative burst, neutrophils switch from glycolysis-dominant metabolism to oxidative PPP, and this reconfiguration maximizes the NADPH yield to fuel superoxide production via NADPH oxidase. In comparison to normal cells, cancer cells show higher levels of intracellular ROS [52] that can increase the rate of pro-oncogenic mutations and facilitate pro-tumorigenic signaling cascades and may also render cancer cells more vulnerable to energetic and oxidative stress. Thus, oxidative PPP in cancer cells is necessary to generate high levels of NADPH to counteract ROS.

Overall, the PPP depends on glucose availability, and when its level is not sufficient, the reduced concentration of NADPH may increase intracellular ROS production. Therefore, alternative glucose-independent mechanisms to generate NADPH are induced. Significantly, in FPR2-stimulated cells, our metabolic analysis detected an increased concentration of malate (Figure 1A), a metabolite that, by exiting the TCA cycle, can produce pyruvate and NADPH in a reaction catalyzed by the malic enzyme.

The non-oxidative PPP consists of several reversible reactions that recruit glycolytic intermediates that can be converted into pentose phosphates and vice versa. Transketolases (TKT) and transaldolases (TALDO) are crucial enzymes of this pathway. Indeed, due to the reversible nature of these enzymes, they can determine the direction of metabolite flux in the non-oxidative PPP. Noteworthy cancer cells can increase the non-oxidative PPP by elevating TKT and TALDO expression [46,53]. Indeed, when the metabolic need for nucleotides exceeds that of NADPH, TKT and TALDO deviate glyceraldehyde-3-phosphate and fructose-6-phosphate from glycolysis to the non-oxidative PPP to generate additional ribonucleotides. Thereby, cancer cells utilize the non-oxidative PPP to generate ribonucleotides for the de novo synthesis of RNA and DNA.

Interestingly, in CaLu-6 cells stimulated with WKYMVm or ANXA1, we measured a time-dependent increase in TKT activity (Figure 2E,G) that was prevented by preincubation with WRW4 (Figure 2F,H). These results strongly indicate that FPR2 signaling activates non-oxidative PPP to fuel RNA and DNA synthesis in cancer cells.

### 3.3. FPR2 Signaling Modulates ASCT2 Expression

Tumor cells show an enhanced demand for amino acids due to their rapid proliferation rate and reconfigure the amino acid metabolism to satisfy protein synthesis and energy demand. The prerequisite for amino acid utilization is the presence of specific cell membrane transporters, and many of these are over-expressed in several types of cancer [54]. In the impaired metabolic conditions of tumor cells, some amino acids are more frequently used than others. 

Glutamine is the most abundant amino acid [55] and contributes to every metabolic task of proliferating tumor cells. In fact, high levels of glutamine provide an available source of carbon and nitrogen atoms for cancer cells to support the biosynthesis of macromolecules, energetics processes, and cellular homeostasis [56]. Many membrane proteins are involved in the transport of glutamine into cells [57], such as the heavily studied solute carrier family 1 neutral amino acid transporter member 5 (SLC1A5; also known as ASCT2) [58].

**Figure 3:**
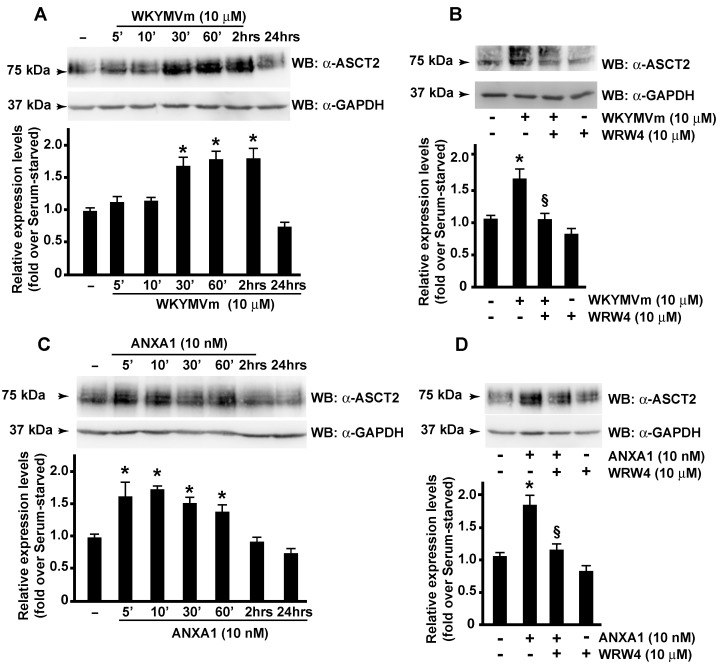
**FPR2 stimulation induces ASCT2 expression.** Growth-arrested CaLu-6 cells were stimulated for the indicated times with WKYMVm (Panel **A**) or ANXA1 (Panel **C**) or preincubated with WRW4 before stimulation (Panels **B**,**D**). Fifty micrograms of whole lysates were resolved on 10% SDS-PAGE and incubated with an anti-ASCT2 (α-ASCT2) antibody. An anti-GAPDH (α-GAPDH) antibody was used as a control for protein loading. Bar graphs are representative of three independent experiments. * *p* < 0.05 compared to unstimulated cells. § *p* < 0.05 compared to stimulated cells.

The export of glutamine out of the cell is mediated by antiporters in exchange for other amino acids, such as leucine, through LAT1, a heterodimer of SLC7A5 and SLC3A2, [59]. ASCT2 is upregulated in many types of cancer by promoting cell growth [60], and ASCT2 over-expression correlates with increased glutamine uptake [61]. In our cells, we observed that WKYMVm or ANXA1 stimulation induced a time-dependent increase in ASCT2 (Figure 3A,C) that was prevented by preincubation with the FPR2 antagonist (Figure 3B,D). 

**Figure 4:**
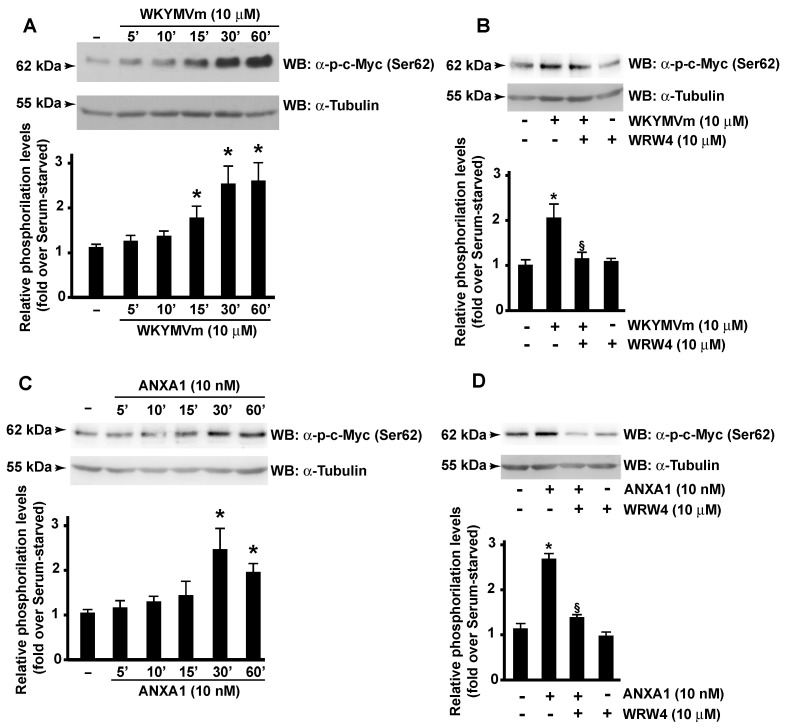
**WKYMVm or ANXA1 stimulation triggers c-Myc phosphorylation.** Growing CaLu-6 cells were serum-deprived for 24 h and then stimulated with WKYMVm (Panel **A**) or ANXA1 (Panel **C**) for the indicated times. Cells were also preincubated with WRW4 before the stimulation with WKYMVm (Panel **B**) or ANXA1 (Panel **D**). Sixty micrograms of whole lysates were resolved on 10% SDS-PAGE and incubated with an anti-phospho-c-Myc (Ser62) (α-p-c-Myc (Ser62)) antibody. An anti-Tubulin (α-Tubulin) antibody was used as a control for protein loading. Bar graphs are representative of three independent experiments. * *p* < 0.05 compared to unstimulated cells. § *p* < 0.05 compared to stimulated cells.

The molecular mechanisms regulating ASCT2 activity are poorly elucidated. Its regulation by miR-137 and by the endoplasmic-reticulum-associated E3 ubiquitin ligase RNF5nd has been observed in several types of cancer [62,63]. Furthermore, discoid protein domain receptor 1, a special type of transmembrane receptor tyrosine kinase, interacts with SLC1A5 and regulates its stability [64]. Finally, the over-expression of c-Myc induces the glutamine addiction of cancer cells [65], thus promoting survival and proliferation [66]. The amount of c-Myc correlates with SLC1A5 expression, and c-Myc directly upregulates SLC1A5, leading to a greater uptake of amino acids [67]. Interestingly, FPR2 localizes in nuclear fractions of CaLu-6 and AGS cells, and nuclear FPR2 activation prompts a decreased Gαi-FPR2 association and triggers ERKs, c-Jun, and c-Myc activation [23]. In response to growth-stimulatory signals, c-Myc protein is phosphorylated at the Ser^62^ residue, which results in its stabilization [68]. In agreement, we detected a time-dependent increase in c-Myc phosphorylation at the Ser^62^ residue in WKYMVm- or ANXA1-stimulated CaLu-6 cells (Figure 4A,C) that was prevented by FPR2 antagonist pretreatment (Figure 4B,D). These results demonstrate that FPR2 signaling, by controlling c-Myc activation, participates in the transcriptional regulation of ASCT2.

### 3.4. FPR2 Signaling Regulates De Novo Synthesis of Pyrimidine Nucleotides

In FPR2-stimulated CaLu-6 cells, we observed increases in aspartate, glutamine, UMP, and CMP concentrations (Figure 1B,C). Aspartate provides three of the four carbon atoms and one nitrogen atom in the biosynthesis of pyrimidine nucleotides. The second nitrogen atom is supplied by glutamine. UMP represents an intermediate product in the de novo synthesis of pyrimidines and can be further phosphorylated to UDP and UTP. CTP synthetase converts UTP into CTP in an ATP-dependent reaction that uses glutamine as an amine donor. CTP can be dephosphorylated into CDP and CMP. Otherwise, UDP and CDP can be deoxygenated into dUDP and dCDP, respectively, by ribonucleotide reductase and further phosphorylated [69].

**Figure 5:**
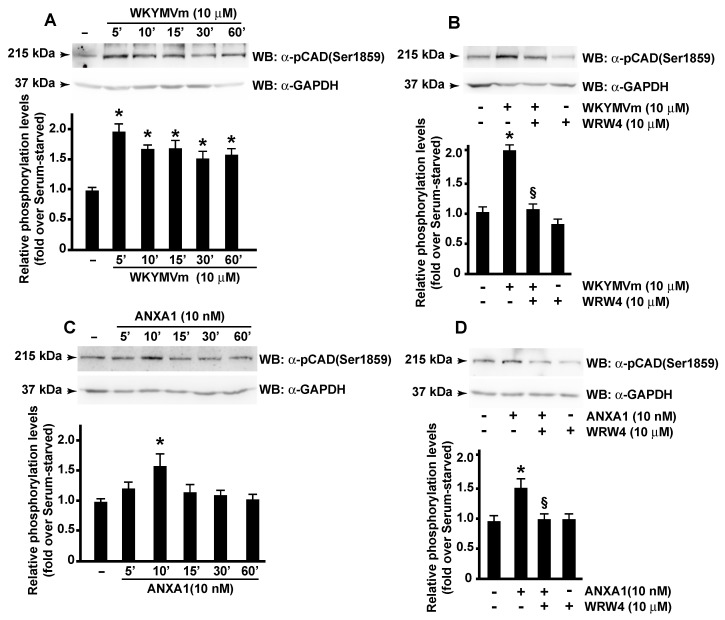
**FPR2 signaling elicits CAD activation.** Serum-deprived CaLu-6 cells were exposed for the indicated times to WKYMVm (Panel **A**) or ANXA1 (Panel **C**). Growth-arrested cells were also preincubated with WRW4 before WKYMVm (Panel **B**) or ANXA1 (Panel **D**) stimulation. Sixty micrograms of whole lysates were resolved on 10% SDS-PAGE and incubated with an anti-phospho-CAD (Ser1859) (α-p-CAD (Ser1859)) antibody. An anti-GAPDH (α-GAPDH) antibody was used as a control for protein loading. Data are representative of three independent experiments. * *p* < 0.05 compared to unstimulated cells. § *p* < 0.05 compared to stimulated cells.

Carbamoyl-phosphate synthetase 2, aspartate transcarbamylase, and dihydroorotase (CAD) form a multifunctional enzyme that participates in the three initial speed-limiting steps of the de novo synthesis of pyrimidine nucleotides in mammals; it is capable of supplying all pyrimidine ribonucleotides and deoxyribonucleotides for RNA and DNA biosynthesis. In resting cells, CAD is mainly localized in the cytoplasm. Its translocation to the nucleus occurs as a result of the entry of the cells into S phase by MAPK that phosphorylate CAD at the Thr^456^ residue. As cells exit S phase, CAD is dephosphorylated at this residue and phosphorylated at Ser^1406^ by PKA, returning the pathway to its basal activity [70]. The phosphorylation at the Ser^1859^ residue on CAD by S6 kinase 1 (S6K1), a downstream ribosomal protein target of mTORC1, stimulates the first three steps of the de novo pyrimidine synthesis and thus helps to advance the cells’ overall progression through S phase of the cell cycle [71,72]. Therefore, we analyzed the Ser^1859^ phosphorylation of CAD in FPR2-stimulated cells, and we observed that either WKYMVm or ANXA1 induced a time-dependent increase in phospho-CAD (Figure 5A,C). Preincubation with WRW4 before stimulation with the two agonists prevented CAD activation (Figure 5B,D), proving that it depends on FPR2 signaling.

**Figure 6:**
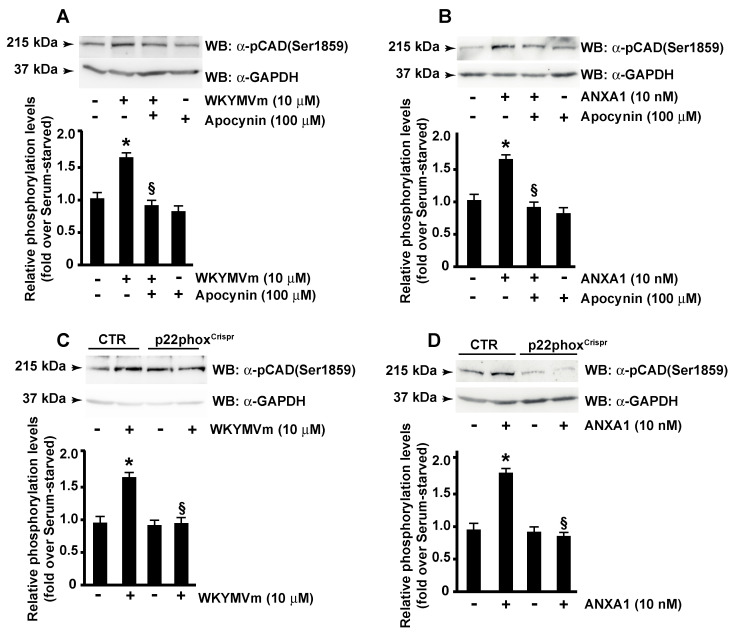
**FPR2-mediated CAD phosphorylation requires NADPH oxidase activation.** Serum-deprived CaLu-6 cells were preincubated with apocynin before the stimulation with WKYMVm (Panel **A**) or ANXA1 (Panel **B**). CaLu-6-control^Crispr/Cas9^ (CTR) and p22phox^Crispr/Cas9^ CaLu-6 (p22phox^Crispr^) cells were serum-starved for 24 h and then stimulated with WKYMVm (Panel **C**) or ANXA1 (Panel **D**). Sixty micrograms of whole lysates were resolved on 10% SDS-PAGE and incubated with an anti-phospho-CAD (Ser1859) (α-p-CAD (Ser1859)) antibody. An anti-GAPDH (α-GAPDH) antibody was used as a control for protein loading. Data are representative of three independent experiments. * *p* < 0.05 compared to unstimulated cells. § *p* < 0.05 compared to stimulated cells.

S6K1 activity is finely regulated. Redox-sensitive mechanisms control the phosphorylation of S6K1, the interaction between S6K1 and mTORC1, and the kinase activity of the S6K1-mTORC1 complex [73]. Since, in several cell types, FPR2 induces NADPH oxidase activity [20,21,30,32,39,74,75,76], we analyzed the ability of NADPH oxidase-dependent ROS production to regulate CAD phosphorylation at the Ser^1859^ residue. Cells were preincubated with apocynin, a potent and selective inhibitor of NADPH oxidase, and exposed to WKYMVm (Figure 6A) or ANXA1 (Figure 6B). The results showed that this treatment prevented CAD activation. By transfecting CaLu-6 cells with a double nickase plasmid, we generated a p22^phox^ knockout cell line [20]. Since p22^phox^ is an essential component of the NADPH oxidase complex, these cells are not able to generate ROS. The stimulation of p22phox^Crispr/Cas9^ CaLu-6 cells with either WKYMVm or ANXA1 did not induce CAD phosphorylation at the Ser^1859^ residue (Figure 6C,D), thus highlighting the role of NADPH oxidase activity on the redox-sensitive mechanisms regulating the mTORC1/S6K1/CAD cascade.

### 3.5. FPR2 Signaling Regulates TCA 

**Figure 7:**
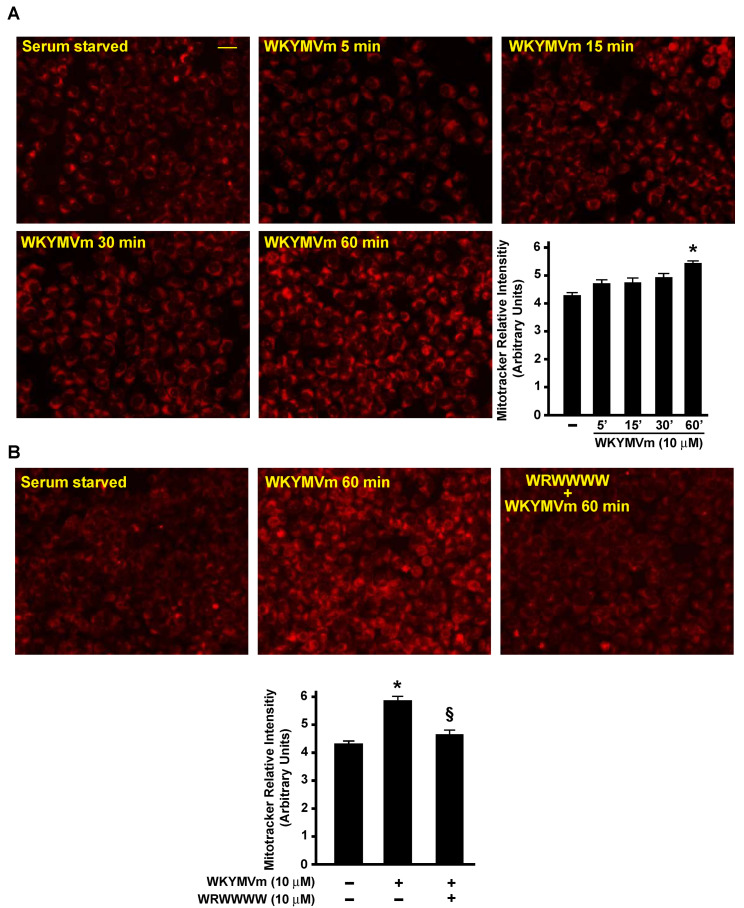
**FPR2 stimulation induces changes in mitochondrial membrane potential.** Serum-starved CaLu-6 cells were stimulated with WKYMVm for 5, 15, 30, or 60 min (Panel **A**) or preincubated with WRW4 before stimulation (Panel **B**). Cells were incubated in the presence of the probe, and mitochondrial fluorescence was measured in a Perkin Elmer Envision 2105 Multiplate reader (Perkin Elmer) using the built-in monochromator. The total number of cells in each well was used for normalization. The results are the means of three independent experiments, and in each separate experiment, every experimental point was analyzed in triplicate. * *p* < 0.05 compared to unstimulated cells. § *p* < 0.05 compared to WKYMVm-stimulated cells.

We observed that the FPR2 stimulation of CaLu-6 cells induced an increase in the glutamate concentration (Figure 1C). Upon entry into the cell via SLC1A5/ASCT2, glutamine is converted by glutaminases to an ammonium ion and glutamate, which is further catabolized through two different pathways. In fact, glutamate can be converted to α-ketoglutarate (αKG) either through reactions catalyzed by glutamate dehydrogenase or by aminotransferases. The byproduct of glutamate dehydrogenase is NH_4_^+^, whereas the byproducts of aminotransferases are other amino acids. αKG can fuel the TCA cycle to generate ATP through NADH and FADH2 production and to generate citrate, thus supporting the synthesis of acetyl-CoA and, in turn, lipids. Accordingly, we found an increase in citrate concentration in FPR2-stimulated cells (Figure 1C); moreover, we observed that WKYMVm stimulation induced time-dependent changes in the mitochondrial membrane potential, as determined by a MitoTracker analysis (Figure 7A). Preincubation with WRW4 prevented these changes, indicating that they depended on FPR2 signaling (Figure 7B).

## 4. Conclusions

**Figure 8:**
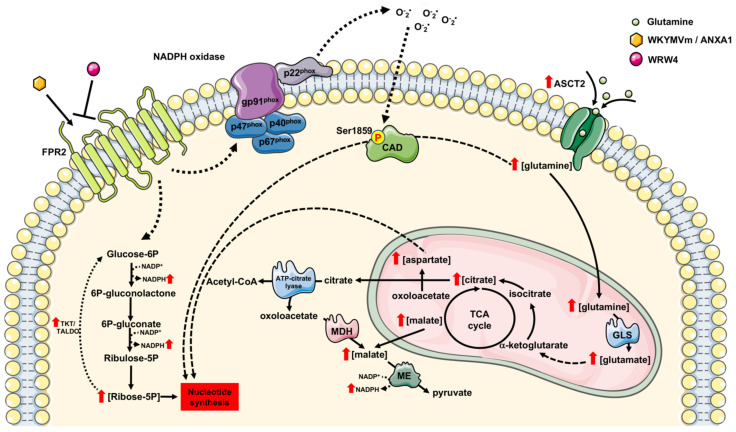
FPR2 signaling triggers metabolic reprogramming of lung cancer cells. FPR2 stimulation increases the ribose 5-phosphate (Ribose-5P) concentration and stim-ulates the pentose phosphate pathway (PPP), thus promoting NADPH generation. WKYMVm and ANXA1 binding to FPR2 also induce enhanced transketolase (TKT) ac-tivity in the non-oxidative phase of the PPP and increases in the citrate and malate concentrations. In the cytosol, citrate can be converted in acetyl-CoA and oxaloacetate, and the latter can be converted into malate through malate dehydrogenase (MDH). Malate may contribute to NADPH generation through a reaction catalyzed by the malic enzyme (ME). FPR2 signaling induces an increase in the expression of the gluta-mine transporter ASCT2 and enhances the concentration of aspartate and glutamine, the pyrimidine nucleotide biosynthesis precursors. CAD, the multifunctional enzyme that participates in the three initial speed-limiting steps of de novo pyrimidine nucleo-tide synthesis, is phosphorylated at the Ser1859 residue in a NADPH oxidase-dependent manner upon the binding of WKYMVm and ANXA1 to the receptor. Therefore, the FPR2-mediated activation of CAD and enhanced concentrations of Ribose-5P, aspar-tate, and glutamine contribute to an increased de novo biosynthesis of nucleotides. Glutamine can be converted into glutamate by glutaminase (GLS). Glutamate can be converted into oxoalacetate and fuel the tricarboxylic acid cycle (TCA) that, in turn, increases the mitochondrial membrane potential. ME: Malic enzyme; MDH: Malate dehydrogenase; TKT: Transketolase; TALD: Transaldolase; GLS: Glutaminase; CAD: Carbamoyl-phosphate synthetase 2, aspartate transcarbamoylase, and dihydroorotase; Glucose-6P: Glucose 6 phosphate; 6P-gluconolactone: 6 phospho-gluconolactone; 6P-gluconate: 6-phospho-gluconate; Ribulose-5P: Ribulose 5-Phosphate; Ribose-5P: Ri-bose 5-phosphate.

This study demonstrates the contribution of FPR2 to the cellular metabolic activities of proliferating cells. Metabolomic profiling revealed that FPR2 signaling (i) promotes PPP and, in turn, NADPH production; (ii) activates the non-oxidative phase of PPP; (iii) induces ASCT2 glutamine transporter expression and, in turn, can contribute to glutamine uptake and metabolism; (iv) regulates TCA; and (v) induces the ROS-dependent de novo synthesis of pyrimidine nucleotides (Figure 8).

Our metabolic profiling revealed that FPR2 enhances the concentrations of both pyrimidine nucleotides and its precursors. Furthermore, our results show that FPR2 signaling induces the activation of CAD, the multifunctional enzyme that participates in the three initial speed-limiting steps of the de novo synthesis of pyrimidine nucleotides. FPR2 triggers the mTORC1/S6K1-dependent phosphorylation of CAD at the Ser^1859^ residue, thus helping to advance the cells’ overall progression through S phase of the cell cycle. The blocking of NADPH oxidase functions prevents CAD phosphorylation, thus proving that it is regulated by redox mechanisms (Figure 8). 

TKT and TALDO are the two major enzymes involved in the non-oxidative PPP and catalyze the formation of glyceraldehyde-3-phosphate and fructose 6-phosphate. In FPR2-stimulated cells, we found increases in Ribose-5P as well as TKT activity (Figure 8). 

Glutamine is the most abundant amino acid, and it provides an available source of carbon and nitrogen for cancer cells to support biosynthesis, energetics processes, and cellular homeostasis. ASCT2, a glutamine transporter, is upregulated in many types of cancer, and its over-expression correlates with increased glutamine uptake. Our results show that FPR2 signaling induces a time-dependent increase in ASCT2 expression (Figure 8) and that c-Myc is involved in the transcriptional regulation of ASCT2. Glutamine plays a critical role in mitochondrial metabolism, and a transcript variant of the SLC1A5 gene has been identified as the mitochondrial glutamine transporter [77]. Glutamine is converted by glutaminases to glutamate, which can be processed to αKG either in reactions catalyzed by glutamate dehydrogenase or by aminotransferases (Figure 8). αKG can fuel the TCA cycle to generate ATP and citrate, thus supporting the synthesis of acetyl-CoA. Accordingly, we observed an increase in the citrate concentration (Figure 1A) and changes in the mitochondrial membrane potential (Figure 7A) in FPR2-stimulated cells. Malate can be exported from TCA to the cytosol and to generate pyruvate and NADPH in a reaction catalyzed by the malic enzyme. Oxaloacetate is converted to aspartate to support nucleotide synthesis (Figure 8). 

Taken together, these results highlight the role of FPR2 in the metabolic reprogramming of cancer cells and suggest FPR2 as a promising therapeutic target for the treatment of human cancers.

FPR2 stimulation increases the ribose 5-phosphate (Ribose-5P) concentration and stimulates the pentose phosphate pathway (PPP), thus promoting NADPH generation. WKYMVm and ANXA1 binding to FPR2 also induce enhanced transketolase (TKT) activity in the non-oxidative phase of the PPP and increases in the citrate and malate concentrations. In the cytosol, citrate can be converted in acetyl-CoA and oxaloacetate, and the latter can be converted into malate through malate dehydrogenase (MDH). Malate may contribute to NADPH generation through a reaction catalyzed by the malic enzyme (ME). FPR2 signaling induces an increase in the expression of the glutamine transporter ASCT2 and enhances the concentration of aspartate and glutamine, the pyrimidine nucleotide biosynthesis precursors. CAD, the multifunctional enzyme that participates in the three initial speed-limiting steps of de novo pyrimidine nucleotide synthesis, is phosphorylated at the Ser^1859^ residue in a NADPH oxidase-dependent manner upon the binding of WKYMVm and ANXA1 to the receptor. Therefore, the FPR2-mediated activation of CAD and enhanced concentrations of Ribose-5P, aspartate, and glutamine contribute to an increased de novo biosynthesis of nucleotides. Glutamine can be converted into glutamate by glutaminase (GLS). Glutamate can be converted into oxoalacetate and fuel the tricarboxylic acid cycle (TCA) that, in turn, increases the mitochondrial membrane potential. ME: Malic enzyme; MDH: Malate dehydrogenase; TKT: Transketolase; TALD: Transaldolase; GLS: Glutaminase; CAD: Carbamoyl-phosphate synthetase 2, aspartate transcarbamoylase, and dihydroorotase; Glucose-6P: Glucose 6 phosphate; 6P-gluconolactone: 6 phospho-gluconolactone; 6P-gluconate: 6-phospho-gluconate; Ribulose-5P: Ribulose 5-Phosphate; Ribose-5P: Ribose 5-phosphate.

## Data Availability

The data presented in this study are available within the article. Other data that support the findings of this study are available upon request to the corresponding authors.
